# Dengue Virus Type 3, Brazil, 2002

**DOI:** 10.3201/eid1109.041043

**Published:** 2005-09

**Authors:** Rita Maria Ribeiro Nogueira, Hermann Gonçalves Schatzmayr, Ana Maria Bispo de Filippis, Flávia Barreto dos Santos, Rivaldo Venâncio da Cunha, Janice Oliveira Coelho, Luiz José de Souza, Flávia Ramos Guimarães, Eliane Saraiva Machado de Araújo, Thatiane Santos De Simone, Meri Baran, Gualberto Teixeira, Marize Pereira Miagostovich

**Affiliations:** * Instituto Oswaldo Cruz, Rio de Janeiro, Brasil;; † Faculdade de Medicina de Mato Grosso do Sul, Campo Grande, Brazil;; ‡ Instituto de Pesquisa Clínica Evandro Chagas, Rio de Janeiro, Brazil;; § Centro de Referencia de Dengue, Campos dos Goytacazes, Brazil;; ¶ Secretaria Municipal de Saúde do Rio de Janeiro, Rio de Janeiro, Brazil;; # Secretaria de Saúde do Estado do Rio de Janeiro, Rio de Janeiro, Brazil

**Keywords:** DENV-3, epidemic, Rio de Janeiro, research

## Abstract

During the summer of 2002, Rio de Janeiro had a large epidemic of dengue fever; 288,245 cases were reported. A subset of 1,831 dengue hemorrhagic fever cases occurred. In this study, performed in the first half of 2002, samples from 1,559 patients with suspected cases of dengue infection were analyzed. From this total, 1,497 were obtained from patients with nonfatal cases, and 62 were obtained from patients with fatal cases. By the use of different methods, 831 (53.3%) cases, including 40 fatal cases, were confirmed as dengue infection. When virus identification was successful, dengue virus type 3 (DENV-3) was obtained in 99% of cases. Neurologic involvement was shown in 1 patient with encephalitis, confirmed by the detection of DENV-3 RNA in the cerebrospinal fluid. This explosive epidemic of DENV-3 was the most severe dengue epidemic reported in Brazil since dengue viruses were introduced in 1986.

## Abstract 2

An explosive epidemic of DENV-3 in 2002 was the most severe dengue epidemic reported in Brazil since dengue viruses were introduced.

Many factors were responsible for the resurgence of epidemic dengue fever (DF) and dengue hemorrhagic fever (DHF) in the final years of the 20th century. Demographic and societal changes such as population growth, urbanization, and modern transportation contributed greatly to the increased incidence and geographic spread of dengue activity (1). The prevalence of the disease is highest in tropical areas of Asia and the Americas, with ≈50–100 million cases of DF and 250,000–500,000 cases of DHF occurring annually worldwide (1–3).

The current epidemiologic situation in Latin America resembles that in Southeast Asia some years ago, with the cocirculation of multiple serotypes in many countries and an increased number of DF and DHF cases. During 2002, Latin American countries reported >1 million cases of DF with >17,000 cases of DHF including 225 deaths (2).

In Brazil, the introduction of dengue virus type 1 (DENV-1) and dengue virus type 2 (DENV-2) in the state of Rio de Janeiro in 1986 and 1990, respectively, resulted in the subsequent spread of these serotypes throughout the country (4). The reintroduction of dengue virus type 3 (DENV-3) in the American continent in 1994 (5) and its rapid spread to the Caribbean Islands in subsequent years (6) resulted in intensified virologic surveillance in the State of Rio de Janeiro, as a response to an imminent threat of DENV-3 epidemics in Brazil. DENV-3 was first isolated in December 2000 in the municipality of Nova Iguaçu, metropolitan region, from a patient with classic DF (7) and initiated a period of cocirculation of DENV-1, DENV-2, and DENV-3 in the state (8). In January 2002, a sudden increase in the number of dengue cases occurred in susceptible populations that had only experienced DENV-1 and DENV-2 epidemics. In the first half of the year, the state reported 288,245 dengue cases, including 1,831 DHF cases and 91 deaths. The metropolitan region including Rio de Janeiro city and surrounding counties reported 246,803 cases and 83 deaths. The number of DHF cases exceeded the total number of cases reported in Brazil from 1986 to the time of the epidemic, and the annual incidence of dengue infection in 2002 in the state reached 1,735 per 100,000 inhabitants (9). We describe laboratory and clinical findings from 1,559 patients, including 62 who died during the largest and most severe epidemic that has occurred in Rio de Janeiro since DENV became endemic in the country in 1986.

## Materials and Methods

### Study Population

The 1,559 case-patients included in this study had acute febrile illness with >2 of the following manifestations: headache, retrobulbar pain, myalgia, arthralgia, rash, and hemorrhagic manifestations. A total of 1,497 cases were in outpatients from different healthcare centers, and the remaining 62 were suspected dengue fatal cases in patients hospitalized in private and public hospitals in the metropolitan area of Rio de Janeiro city. The age range (1–73 years) was quite evenly distributed; 10.5% were 1–10 years of age, and 16.9%–19.9% of all patients were in each 10-year age group.

### Laboratory Methods

Acute-phase serum specimens, cerebrospinal fluid (CSF), and fresh tissues were stored at –70°C and convalescent-phase serum specimens at –20°C until tested. Dengue infections were confirmed by virus isolation or viral RNA detection by reverse-transcriptase polymerase chain reaction (RT-PCR), by immunoglobulin (Ig) M and/or IgG seroconversion, or by the demonstration of DENV antigen in formalized fixed autopsy tissues by immunohistochemical tests.

### Virus Isolation

Virus isolation was performed for all serum samples obtained until day 7 after the onset of disease (n = 927), by infection of clinical specimens into clone C6/36 of *Aedes albopictus* cells. The virus isolates were typed by the indirect fluorescent antibody test with serotype-specific monoclonal antibodies (10).

### RNA Extraction and RT-PCR

RT-PCR (11) was performed as a rapid molecular tool to detect and type DENV only in acute-phase sera and fresh tissues from patients who died, hospitalized patients, and outpatients whose disease severity was characterized by thrombocytopenia, hemorrhagic manifestations, or both (n = 282). Viral RNA was extracted from clinical samples (sera, CSF, and tissue) with QIAamp Viral RNA Mini Kits (Qiagen, Inc., Valencia, CA, USA) according to the manufacturer's protocol.

### Serology

Dengue IgM-capture enzyme-linked immunosorbent assay (ELISA) (PanBio, Brisbane, Australia) was performed according to the manufacturers' instructions in sera obtained after day 5 after onset of disease and in all sera from patients who died (n = 1,060). An in-house IgM antigen capture ELISA (MAC-ELISA) (12) was also performed to confirm dengue infection in sera from patients who died.

IgG-ELISA was performed, as previously described (13), in serum samples available from patients with fatal outcomes (n = 37) and in paired serum samples from patients with fatal cases (n = 88). According to the IgG-ELISA criteria, the immune response is defined as primary when acute-phase serum samples obtained before day 5 of illness have IgG antibody titers <1:160 and convalescent-phase sera have titers <1:40,960. Infections are considered secondary when IgG titers are >1:160 in the acute-phase serum and >1:163,840 in convalescent-phase samples.

### Immunohistochemical Procedure

Sections of formalin-fixed, paraffin-embedded tissues were processed by using the streptavidin-biotin method, according to the manufacturer's protocol (Kit LSAB, DAKO, Carpinteria, CA, USA). Monoclonal antibodies for DENV-1, -2, and -3 were provided by the Centers for Disease Control and Prevention.

## Results

### Laboratory Findings

DENV was isolated from 237 (25.6%) of 927 acute-phase serum specimens injected into C6/36 cells and identified as DENV-3 (n = 234), DENV-1 (n = 2), and DENV-2 (n = 1). Of the 927 serum samples, 282 were submitted for virus isolation and RT-PCR. RT-PCR identified 129 (45.7%) of 282 cases as DENV-3. Thus, the overall results obtained with both methods showed that 321 (99.1%) of 324 viruses identified were DENV-3. A total of 171 samples were submitted for both MAC-ELISA and either virus isolation or RT-PCR. When MAC-ELISA results were added to the diagnostic algorithms, case confirmation reached 53.3% (831/1,559) (Table 1).

**Table 1:** Monthly distribution of suspected dengue cases investigated January–July, 2002, State of Rio de Janeiro*

Month	Virus isolation positive/studied (%)	RT-PCR positive/studied (%)	Serotype detected	MAC-ELISA positive/studied (%)	IgG-ELISA positive/studied (%)	Confirmed cases/studied cases (%)	Deaths positive/studied (%)
January	114/360	47/93	2 DENV-1; 1 DENV-2; 135 DENV-3	203/373	61/67	308/525	6/8
February	61/315	49/89	103 DENV-3	212/356	29/41	279/504	15/20
March	55/173	28/69	72 DENV-3	123/220	15/29	187/375	15/23
April	3/45	5/18	7 DENV-3	38/71	3/8	49/97	4/8
May	2/22	0/7	2 DENV-3	4/26	ND	6/38	0/1
June	2/6	0/6	2 DENV-3	0/9	0/2	2/11	0/2
July	0/6	ND	0	0/5	0/1	0/9	0
Total	237/927 (25.6)	129/282 (45.7)	2 DENV-1; 1 DENV-2; 321 DENV-3	580/1,060 (54.7)	108/148 (73.0)	831/1,559 (53.3)	40/62 (64.5)

*RT-PCR, reverse transcriptase–polymerase chain reaction; MAC-ELISA, immunoglobulin M antigen capture enzyme-linked immunosorbent assay; IgG, immunoglobulin G; DENV, dengue virus; ND, not done.

Dengue infection was confirmed in 40 (64.5%) of 62 patients who died. In 21 of these cases, infection was confirmed by at least 2 methods employed as follows: 2 cases by virus isolation and RT-PCR; 9 cases by MAC-ELISA and RT-PCR; 6 cases by RT-PCR and immunohistochemistry; 2 cases by MAC-ELISA and immunohistochemistry; 1 case by virus isolation, RT-PCR, and immunohistochemistry; and 1 case by virus isolation, MAC-ELISA, and RT-PCR.

The male: female ratio was 1:1.08 in DENV-3 patients and 1: 1.6 when only fatal cases were considered. The age range of patients who died was 7–65 years. A total of 103 clinical samples (serum or fresh tissues samples of liver, spleen, lung, kidney, and brain) were available from the 62 patients with fatal outcome. In these samples, we were able to detect viral RNA, by using RT-PCR, in 33 (32.0%) of 103 specimens. DENV-3 RNA was identified from the CSF of 1 patient (Table 2). Of the 99 clinical specimens injected into C6/36 cells, DENV-3 was recovered from 6 specimens; a total of 24 fatal cases were confirmed as DENV-3 infection by using both methods (Table 2).

**Table 2:** Investigation of suspected fatal dengue cases according to available clinical samples*

Clinical specimen	RT-PCR positive/studied (%)	Virus isolation positive/studied (%)	Serotype detected	MAC-ELISA positive/studied	Immunohistochemistry positive/studied	Confirmed cases/studied cases (%)
Serum	15/42	4/38	15 DENV-3	18/42	ND	26/42 (61.9)
CSF	1/ 2	0/2	1 DENV-3	0/2	ND	1/2
Fresh tissues	17/59	2/59	17 DENV-3	ND	ND	17/59(28.8)
Formalin-fixed and paraffin embedded tissues	ND	ND	0	0	23/48	23/48 (47.9)
Total	33/103 (32.0)	6/99 (6.0)	33 DENV-3	18/44 (40.9)	23/48 (47.9)	40/62† (64.5)

*RT-PCR, reverse transcriptase–polymerase chain reaction; MAC-ELISA, immunoglobulin M antigen capture enzyme-linked immunosorbent assay; DENV, dengue virus; IgG, immunoglobulin G; ND, not done; CSF cerebrospinal fluid. †Total of confirmed fatal cases by any method/total of fatal cases studied.

Immunohistochemical procedures detected DENV antigen in 48% of specimens from patients with fatal cases, mainly in hepatocytes. Among all the tissues analyzed, the liver was the site where DENV was most frequently recovered by using RT-PCR, virus isolation, and immunohistochemistry (Table 3). The pattern of immunoreactivity in all tissues showed cytoplasmic granular positivity.

**Table 3:** Dengue virus detection according to tissues samples analyzed from patients with laboratory-confirmed fatal cases*

Tissues sample	RT-PCR positive/studied	Virus isolation positive/studied	Immunohistochemistry positive/studied
Liver	7/7	2/6	15/23
Lung	4/4	0/4	2/10
Brain	3/3	0/3	2/7
Kidney	2/2	0/2	0
Spleen	1/1	0/1	4/11

*RT-PCR, reverse transcriptase–polymerase chain reaction.

The histopathologic findings in patients with confirmed fatal cases showed that the liver was the most affected organ, with macro- and microvacuolization and discrete lymphocytic infiltration of the periportal space. Focal necrosis, swelling of hepatocytes, and colestasis were frequently observed. Edema and congestion were the predominant findings in the brain. Microhemorrhagic foci were also present; however, a marked inflammatory reaction was not observed. Meningeal congestion was frequent. Intraalveolar hemorrhaging was seen in the lungs, associated with the inflammatory infiltration of lymphocytes. In the spleen, congestion of the paracortical zone was the most frequent finding.

IgG-ELISA was performed on 37 serum specimens available from patients who died to characterize the immune response, 20 (54.1%) cases were classified as primary infection, 9 (24.3%) cases as secondary, and 8 (21.6%) cases as inconclusive. In 88 nonfatal cases of confirmed DENV-3 infection, 49 (55.7%) were classified as primary infection and 39 (44.3%) as secondary infection.

### Clinical Findings

When stratified analysis was conducted on data from the 297 DENV-3 patients who died (131 male and 166 female), confirmed by RT-PCR, virus isolation, or both, the following signs and symptoms were noted: fever (100.0%), headache (96.3%), myalgia (80.8%), prostration (71.4%), nausea/vomiting (70.0%), retroorbital pain (58.9%), and arthralgia (54.9%). Hypotension (8.8%) and abdominal pain (1.7%) were also observed in some patients with severe cases. Neurologic signs were observed in 1.3%, and hepatic involvement was demonstrated by the number of patients with jaundice (5.4%). Trombocytopenia was noted in 6.1% of patients. The hemorrhagic manifestations in 297 of these patients were metrorrhagia (13.3%), epistaxis (3.7%), melena (5.1%), hematuria (4.0%), hematemesis (2.7%), bleeding gums (1.3%), hemoptysis (0.7%), and ecchymosis (1.0%).

## Discussion

During 2002, a total of 771,551 dengue cases were reported in Brazil, mainly in the southeastern and northeastern regions. That number corresponded to 80% of reported dengue cases in the Americas (http://www.paho.org; 21 Nov 2002).

The State of Rio de Janeiro, with a total population of 14,391,282 inhabitants, is located in an area of 43,696,054 km^2^ on the coast of the southeast region of Brazil. Most of the population (11,094,994) inhabit the greater metropolitan region of the state, including the capital Rio de Janeiro and another 18 surrounding municipalities. This region caused 308,125 (87.5%) of 351,959 DENV-1 and DENV-2 cases reported in the state in the last 15 years (9).

The introduction of DENV-3 into Rio de Janeiro in 2000 placed the region at high risk for a new epidemic due to this serotype, since the introduction of a new serotype into a susceptible population with high mosquito densities may produce a large epidemic after a lag period (14). Indeed, 1 year after the DENV-3 introduction, this serotype was responsible for the most severe epidemic in the state's history in terms of the highest number of reported cases, the severity of clinical manifestations, and the number of confirmed deaths. In this DENV-3 epidemic, the number of DHF/dengue shock syndrome (DSS) cases (1,831) and deaths (91) exceeded the total number of DHF/DSS cases (1,621) and deaths (76) in the entire country from 1986 to 2001 (15). The occurrence of 3 confirmed deaths in children <15 years of age could represent a change in the epidemiologic scenario, since DHF/DSS cases in Brazil have been observed almost exclusively in adults (16).

When we analyzed the clinical data on patients with nonfatal cases, the frequency of fever, headache, and myalgias was similar to those observed during the DENV-1 epidemic in 1986 to 1987 (17); however, prostration, hemorrhagic manifestations, and hypotension were observed more often in the more recent DENV-3 epidemic. Furthermore, prostration caused by DENV-3 infection was previously described as a cause for hospital admission during an epidemic in Queensland, Australia (18). Mild and severe forms of the disease were also reported during DENV-3 epidemics in New Caledonia and Tahiti, respectively (19,20).

An increase in unusual manifestations was observed during this epidemic, characterized by the incidence of central nervous system (CNS) involvement and hepatitis. Although CNS involvement has been previously reported during dengue epidemics, including those in Brazil (21,22), it increased during this epidemic, when many patients reported dizziness. In 1 fatal case, this involvement was confirmed by detecting DENV-3 RNA in CSF. Neurologic disorders associated with dengue cases have been referred to as dengue encephalopathy, attributed to immunopathologic responses and not to CNS infection. However, isolating DENV-3 and detecting DENV-2 by using RT-PCR from CSF provide evidence that DENV has neurovirulent properties and can cause encephalitis in both primary and secondary infections (23). Moreover, the breakdown of the blood-brain barrier has been previously demonstrated in fatal dengue cases (24). Data about transaminase levels from dengue patients were not available; however, the impact of DENV infection on liver functions could be demonstrated by patients with jaundice. Alterations in levels of aspartate aminotransferase and alanine aminotransferase were observed in 63.4% and 45% of dengue patients in a study performed during a DENV-3 outbreak in the city of Campos de Goytacazes in the same year (25). Transient derangement of liver functions has been previously demonstrated in dengue patients and in DHF patients with or without hepatomegaly (26,27). In this study, hepatomegaly was reported only in patients who died. A low rate of hepatomegaly due to dengue infection was previously reported in Manila; 1% of patients with confirmed cases had this sign. These levels are considerably lower than the levels observed in Bangkok (80%–90%) and Jakarta (49%) (26). A study on clinical differences observed in patients with dengue caused by DENV-3 showed that they had 3.06 times more risk for abdominal pain than patients with DENV-1 and 6.07 times more risk for shock than patients infected with DENV-2 (28).

A retrospective study of the patients who died (29) in this epidemic showed that warning signs occurred in 88.1% of patients on hospital admission: hypotension (59.5%), abdominal pain (35.7%), and preshock (35.7%). During hospitalization, the proportion with hypotension reached 75.6% and with shock, 61%. The World Health Organization criteria for DHF were fullfilled by 35.5% of the hospitalized patients. Death due to shock occurred in 57.8% of patients, cardiac failure in 17.8%, and massive pulmonary hemorrhage and meningoencephalitis in 2 cases (29).

Liver tissue was the most important tissue for virus detection by using virus isolation, RT-PCR, or immunohistochemistry. Recently, the liver was recognized as a major target organ in the pathogenesis of DENV infection; the active replication in hepatocytes (30,31) could explain these findings. The virologic confirmation of cases in 24 patients who died was similar to that described in Indonesia (32).

The increased mortality rate has already been related to the general phenomenon of increased dengue incidence and severity. The reintroduction of DENV-3 in Puerto Rico and Queensland did not result in death (14,18); however, in Jakarta the DENV-3 fatality rate was nearly 3 times higher than the fatality rate observed for the other serotypes (33).

In this study, the disease severity and the occurrence of deaths resulting from primary infections could be partially explained by the virulence of the DENV-3 strain. Analysis of the partial nucleotide sequence of the genome showed that Brazilian DENV-3 belongs to genotype III (Sri Lanka/India), similar to the strains currently circulating on the American continent (34). Previous studies have shown that this genotype caused DHF epidemics in Sri Lanka and India and was associated with DHF cases in Mexico (35). Fatal cases resulting from dengue primary infections were described before DENV-3 was introduced in Brazil (36), although the largest number of DHF/DSS cases occurring in the state were due to secondary DENV-2 infections (Southeast Asia-Jamaican genotype) (16). These findings showed that some DENV strains can be more virulent than others and that antibody-dependent enhancement alone does not explain all cases of severe disease (33,37–39). Genotyping studies performed in Sri Lanka and French Polynesia showed that viral strains in themselves are an important risk factor for DHF/DSS (20,40).

The scenario of dengue in Brazil indicates that more emphasis should be placed on efforts to control the vector. An active epidemiologic surveillance laboratory should be supported, and a clearer understanding of the epidemiologic characteristics of dengue transmission is required.
